# Choosing the Path: Insights Into Zambian Medical Students' Specialty Selections

**DOI:** 10.1002/hsr2.70463

**Published:** 2025-02-10

**Authors:** Gerald Musa, Samuel Chilawa, Alick Bwanga, Bupe Mumba Mwela, Laston Chikoya, Bipin Chaurasia

**Affiliations:** ^1^ Peoples' Friendship University of Russia Named After Patrice Lumumba (RUDN) Moscow Russia; ^2^ Michael Sata School of Medicine Copperbelt University Ndola Zambia; ^3^ Department of Surgery University of Zambia School of Medicine Lusaka Zambia; ^4^ Levy Mwanawasa Medical University Lusaka Zambia; ^5^ Department of Neurosurgery Neurosurgery Clinic Birgunj Nepal

**Keywords:** future specialization preferences, medical students, specialization

## Abstract

**Background:**

The specialization of medical students is essential for enhancing healthcare quality, and meeting the diverse needs of patients, with medical training programs significantly influencing their career trajectories and expertise development. This study aimed to investigate the future specialty preferences of medical students from various medical schools in Zambia.

**Materials and Methods:**

The study included participants from seven medical schools who had completed at least their first clinical clerkship. Data collection involved distributing structured questionnaires containing Likert scale items and open‐ended questions via an online Google Sheets platform. Data collected included: demographics, name of medical school, year of study, specialty preferences, factors influencing specialty preferences, awareness about medical specialties, and career pathways.

**Results:**

A study involving 127 medical students in clinical clerkships revealed a gender ratio of 1.1:1 male to female, with 73% aged between 20 and 25 years. Specialty preferences varied, with internal medicine (12%) and cardiovascular surgery (10%) being popular choices, and 62% changing their preferences during clerkships. Key factors influencing specialty choice were personal interest (74%), work‐life balance (41%), and career prospects (30%). Only 21% were very familiar with medical specialties, and 83% rated mentorship availability poorly and suggested organizing workshops to enhance awareness. Future plans included further specialization (54%), international humanitarian work (54%), and medical academics (27%).

**Conclusion:**

Despite recognizing the importance of mentorship, students rated the availability of mentorship and informational resources as inadequate. The findings emphasize the need for enhanced mentorship programs, comprehensive career guidance, and targeted informational workshops to support informed specialty choices, contributing to a better‐prepared and more satisfied medical workforce.

## 1 Introduction

Specialization and career choices of medical doctors have a significant impact on the quality of the healthcare system [1]. The choice of medical specialization is a crucial component of human resources planning, which has received increasing attention in recent years [2, 3]. Training medical professionals to specialize in different medical specialties is a key objective of undergraduate medical education [4]. Specialist training is vital to elevate the quality of medical education and raise the standard of medical care. To accomplish these goals, adequate numbers of professionals in all therapeutic disciplines are therefore required [5]. Selecting a postgraduate career path is a crucial decision that is frequently challenging to change once residency training has begun [6]. Medical students may find it challenging to select a professional path in medical practice due to several confounding factors such as previous specialty exposure during training, job satisfactions, expected income, availability of training opportunities in the country or region, cultural and social values [7, 8]. Understanding medical students' specialty preferences can assist directors of medical programs in creating or enhancing training facilities that prepare students for the desired future [9]. A study done in Nigeria found that top clinical specialties chosen by respondents were obstetrics and gynecology (24.9%), surgery (18.9%), internal medicine (14.1%), and pediatrics (8.1%) while Pathology (2.7%), radiography (1.1%), ophthalmology (4.3%), and ENT (0%) were the least popular specialties [10]. Despite the increasing body of literature on specialty selection among medical students globally, there is a dearth of research specifically focused on low‐middle‐income countries (LMICs) like Zambia where there is a dire need for specialized care [11, 12]. This research analyses the specialty preferences of medical students studying in Zambia and the factors affecting their choice of career path.

## 2 Participants and Methods

### 2.1 General Design

The study employed a cross‐sectional design to investigate the future specialization preferences of Zambian medical students in their 5th, 6th and 7th year of training. The survey methodology was structured in line with the CHERRIES checklist [13], ensuring rigorous reporting of web survey data. The study was conducted between February and June 2024 across nine medical universities in Zambia, including Copperbelt University, Mulungushi University, the University of Zambia, Lusaka Apex Medical University, Levy Mwanawasa Medical University, the University of Lusaka, Eden University, Cavendish University, and Texila American University.

A stratified random sampling technique was utilized to ensure representation from diverse demographic backgrounds and academic settings. Participants were recruited through institutional WhatsApp groups and direct messaging. The online questionnaire, hosted on Google Forms, included built‐in features to prevent multiple submissions, such as unique submission tracking and restrictions on repeat responses.

The questionnaire consisted of 24 questions distributed over six pages, with four questions per page, and was designed to take approximately 5 min to complete. Of the 174 participants who initially agreed to participate, 127 completed the survey, resulting in a response rate of 73%.

### 2.2 Development and Validation of Questionnaire

The questionnaires used in this study were developed based on a comprehensive review of existing literature and input from subject matter experts in medical education and specialty selection. To ensure validity and reliability, a pilot study was conducted with a sample of 15 participants, representative of the target demographic. Feedback from the pilot phase informed refinements to enhance question clarity, relevance, and structure. The questionnaire was assessed for reliability using Cronbach's alpha, yielding a score of 0.82, indicating good reliability for the main domains of the survey. These steps ensured that the final version of the questionnaire was both robust and fit for purpose.

### 2.3 Inclusion and Exclusion Criteria

Medical students who had at least completed their first clinical clerkship were included in the study. Students who had not completed their first clinical clerkship including preclinical students were not included.

### 2.4 Data Collection

Data collection involved the distribution of structured questionnaires containing Likert scale items and open‐ended questions, administered using a structured online Google Sheet. Informed consent was obtained and documented (written) from all participants before data collection. Confidentiality and anonymity of responses were assured and observed to encourage candid responses. Data collected included: gender, age, year of study, clinical clerkship completed, name of medical school, specialty preferences, factors influencing specialty preferences, awareness about medical specialties and career pathways.

### 2.5 Statistical Analysis

After collecting the data, the raw data was checked for completeness, consistency and accuracy and then coded, data entry and analysis were carried out using IBM Statistical Package for Social Sciences (SPSS) Version 26. Data were analyzed using descriptive and inferential statistical methods. Categorical variables, such as gender, age groups, university affiliation, and specialty preferences, were summarized as frequencies and percentages. Associations between variables (e.g., gender and specialty preference, age group and specialty preference) were tested using Pearson's chi‐square test of independence. For variables with small sample sizes, Fisher's exact test was considered where applicable. The relationship between specialty preference (surgical vs. nonsurgical) and demographic characteristics such as age, gender, year of study, and institution was examined. A *p*‐value < 0.05 was considered statistically significant for all tests.

## 3 Results

### 3.1 Demographics

**Figure 1:**
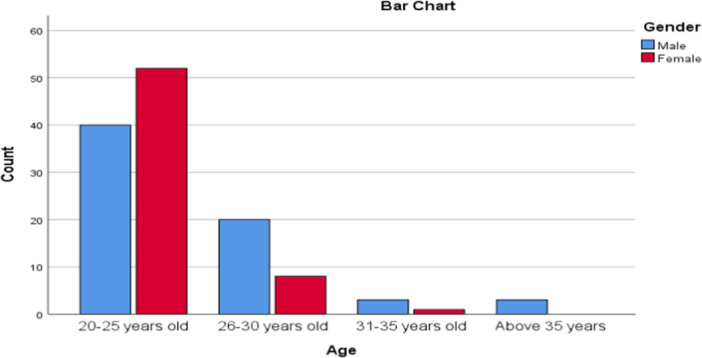
Distribution of participants in difference age groups.

A total of 127 medical students (MS) in clinical clerkships participated in the study, 66 males (52%) and 61 females (48%) with a 1.1:1 male‐to‐female ratio. Seventy‐three percent (92/127) of the MS were aged between 20 and 25 years, 22% (28/127) were aged between 26 and 30 years, 3% (4/127) were 31–35 years, and 2% (3/127) were over 35 years (Figure 1). Thirty‐one (24.4%) participants were from Copperbelt University, 24 (18.9%) Mulungushi University, 24 (18.9%) University of Zambia, 22 (17.3%) from Lusaka Apex Medical University 14 (11%) Levy Mwanawasa Medical University, 8 (6.3%) University of Lusaka, 2 (1.6%) Eden University, 1 (0.8%) Cavendish University and 1 (0.8%) from Texila American University (Table 1).

**Table 1:** Demographics.

Variables		n (%)
Gender	Male	66 (52)
	Female	61 (48)
Age range (years)	< 20	0
	20–25	92 (73)
	26–30	28 (22)
	31–35	4 (3)
	> 35	3 (2)
Institution	The Copperbelt University	31 (24.4)
	Mulungushi University	24 (18.9)
	The University of Zambia	24 (18.9)
	Lusaka Apex Medical University	22 (17.3)
	Levy Mwanawasa Medical	14 (11)
	University of Lusaka	8 (6.3)
	Eden University	1 (0.8)
	Cavendish University	2 (1.6)
	Texila American University	1 (0.8)
Year of Study	5th	60 (47)
	6th	36 (28)
	7th	31 (25)

### 3.2 Specialty Preference

**Figure 2:**
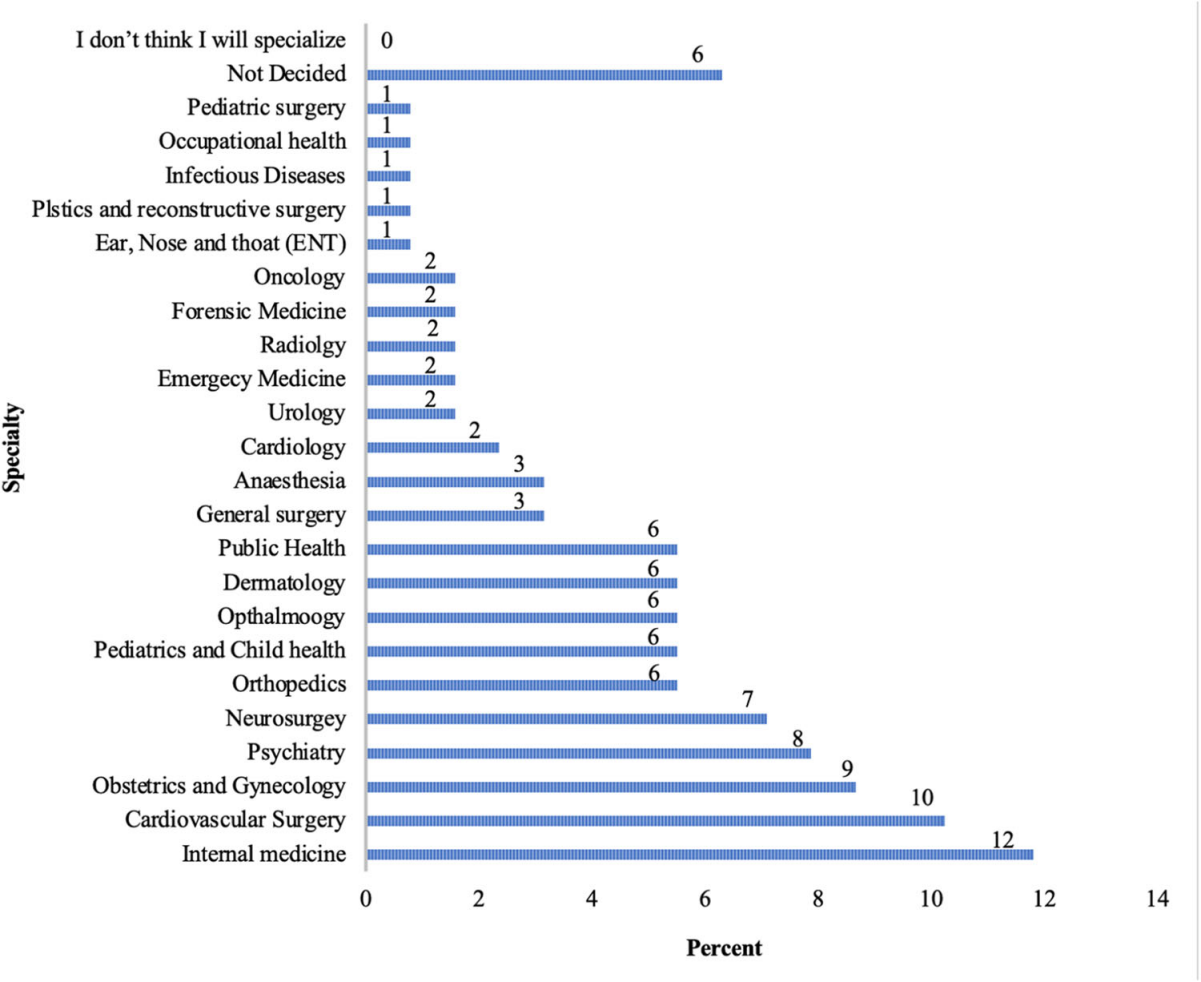
Specialty preference.

**Figure 3:**
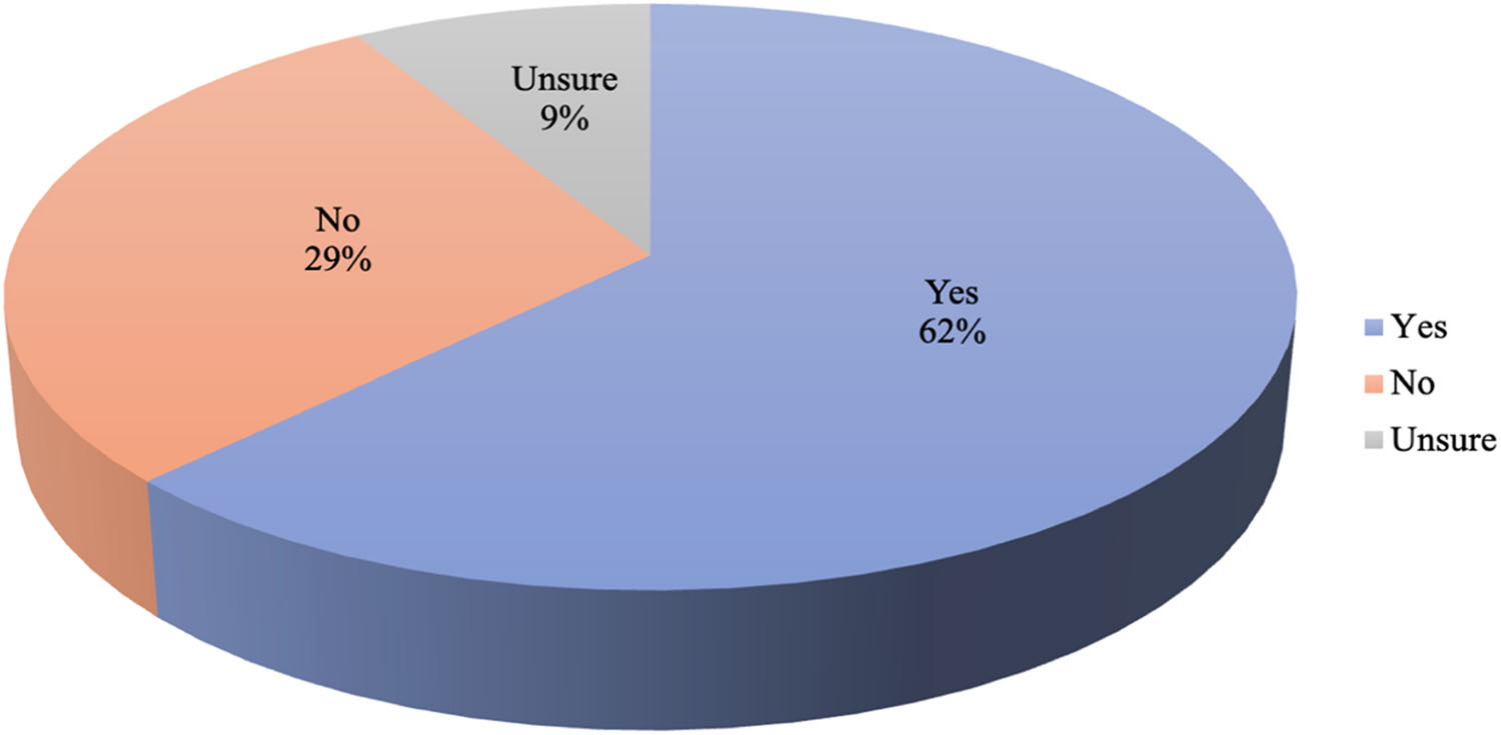
Changes in specialty aspirations.

Six percent of participants are not decided about picking a specialty. Twelve percent picked internal medicine, cardiovascular surgery (10%), obstetrics and gynecology (9%), psychiatry (8%), neurosurgery (7%), 6% each for orthopedics, pediatrics and child health, ophthalmology, dermatology and public health. None of the participants indicated a desire not to specialize (Figure 2). Sixty‐two percent changed their choice of specialty during the clinical clerkship (Figure 3).

**Figure 4:**
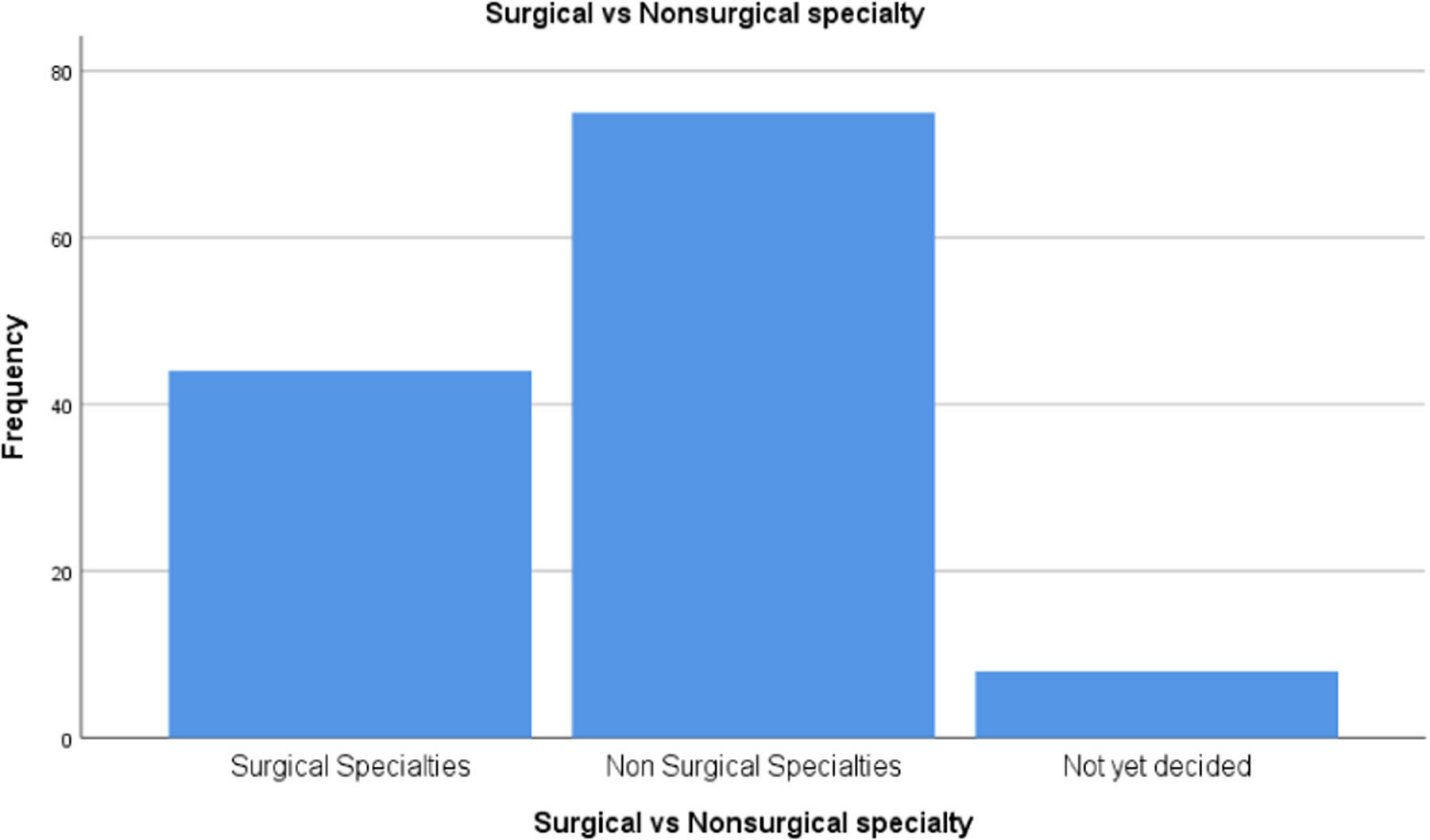
Distribution of participants in surgical and nonsurgical specialties.

The specialties were further divided into surgical (*n* = 44), nonsurgical (*n* = 75) and not decided (*n* = 8) (Figure 4). In the surgical specialty group, there were more males (*n* = 27) than females (*n* = 17), however, the difference was not statistically significant *p* = 0.175 (Table 2).

**Table 2:** Distribution of gender in surgical and nonsurgical specialties.

Gender	Surgical vs nonsurgical specialty			Total
	Surgical	Nonsurgical	Not yet decided	
Male	27	36	3	66
Female	17	39	5	61
Total	44	75	8	127

**Figure 5:**
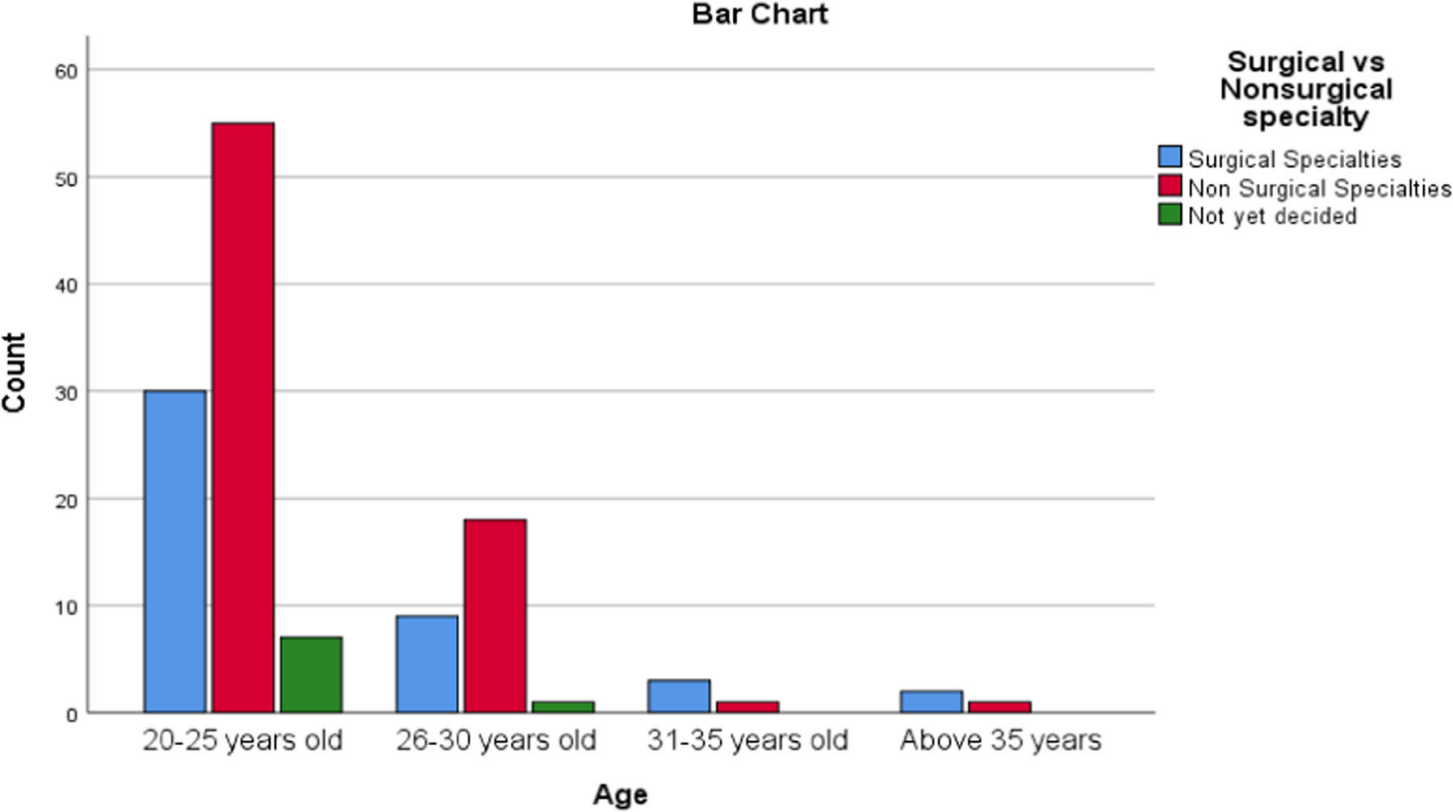
Distribution of participants in different age brackets in surgical and nonsurgical specialties.

On analysis of age and choice of specialty, there was no statistically significant difference between surgical and nonsurgical preference in difference age brackets *p* = 0.18 (Figure 5).

**Figure 6:**
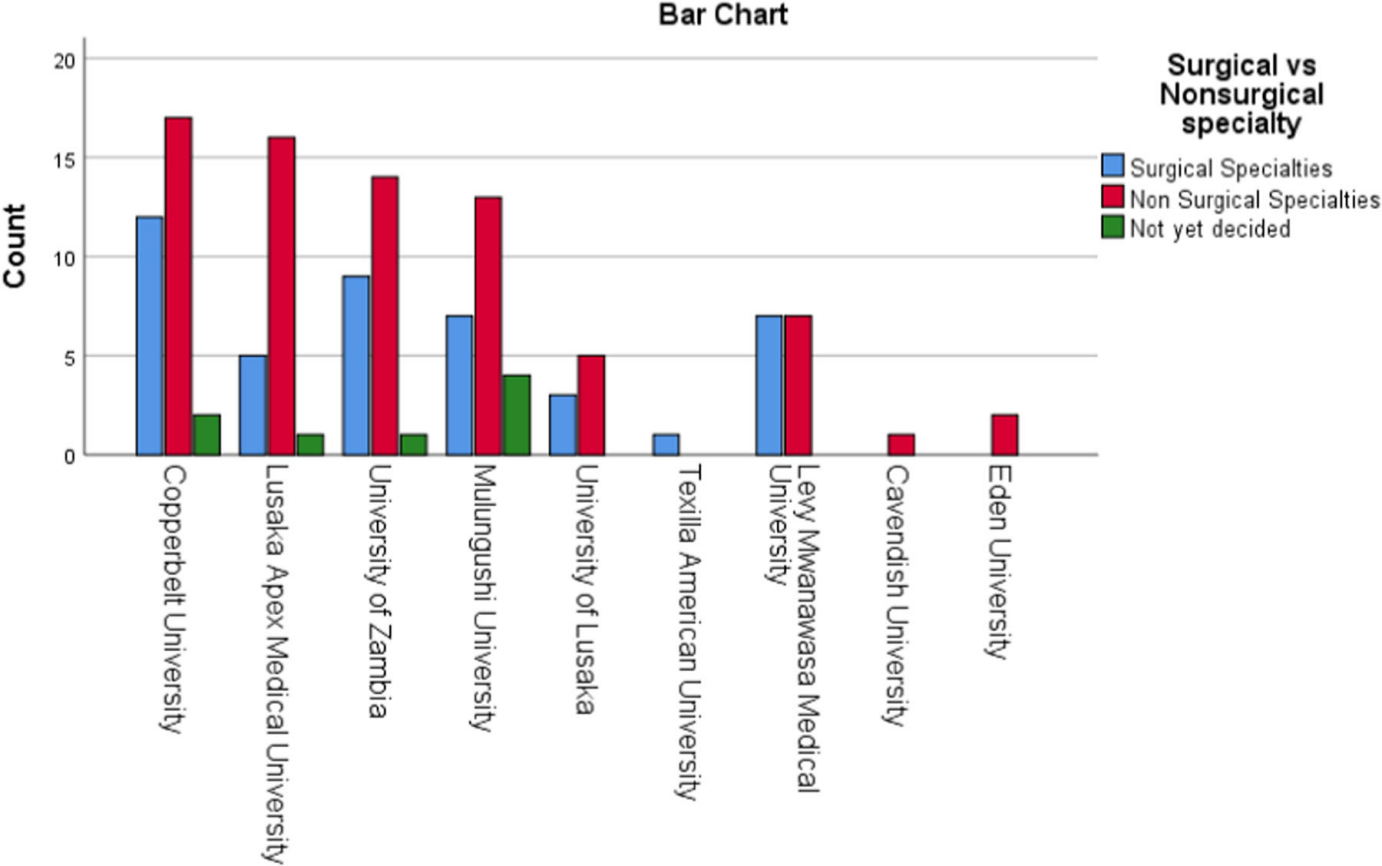
Distribution of participants in different medical institutions in surgical and nonsurgical specialties.

There was no statistically significant difference between surgical and nonsurgical specialty preference in the different years of study or medical institutions *p* = 0.12 and *p* = 0.31 respectively (Table 3 and Figure 6).

**Table 3:** Distribution of participants in different years of study in surgical and nonsurgical specialties.

Year of study	Surgical vs Nonsurgical specialty			Total
	Surgical	Nonsurgical	Not yet decided	
5th year	23	33	5	61
6th year	14	20	1	35
7th year	7	22	2	31
Total	44	75	8	127

### 3.3 Factors Influencing Specialty Selection

**Figure 7:**
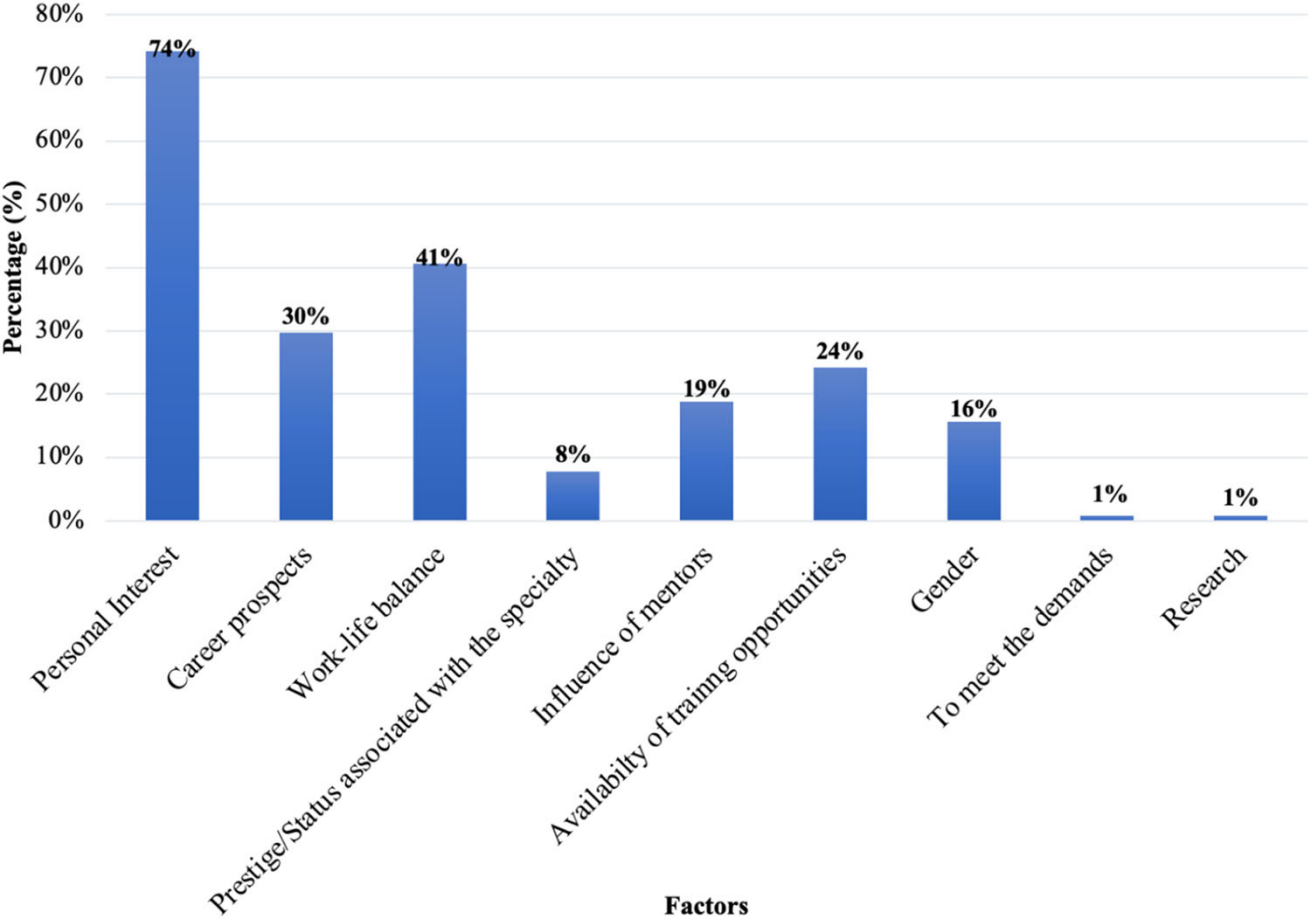
Factors influencing decision regarding specialty selection.

The main factors influencing specialty selection included: personal interest (74%), work‐life balance (41%) and career prospects (30%) (Figure 7). Financial considerations were significant for 41% of respondents, moderate for 36%, minor for 11%, and not a factor for 12%. Mentorship availability was very important for 46% of students, important for 28%, neutral for 21%, and not important for 5%.

When considering the balance between job satisfaction and potential earnings, 47% found it favorable, 37% neutral, 9% unfavorable, and 7% were unsure. Job availability or market saturation was a concern for 54% of respondents (Table 4).

**Table 4:** Influence of financial factors, mentorship, job availability, and work‐life balance on specialty choice.

Variables		n (%)
Role of financial considerations	Significant role	52 (41)
	Moderate role	46 (36)
	Minor role	14 (11)
	Not a factor	15 (12)
Influence of mentorship availability	Very important	58 (46)
	Important	36 (28)
	Neutral	27 (21)
	Not important	6 (5)
Balance between job satisfaction and earnings	Favorable	60 (47)
	Neutral	47 (37)
	Unfavorable	12 (9)
	Not sure	8 (7)
Concerns about job availability/market saturation?	Yes	69 (54)
	No	58 (46)

### 3.4 Level of Awareness About Various Medical Specialties and Career Pathways

Only 21% of participants were very familiar with the various medical specialties available for specialization, 52% were somewhat familiar, and 27% were not very familiar. Regarding career pathways and training requirements, 18% were very aware, 35% somewhat aware, 38% not very aware, and 9% not aware at all. Forty‐eight percent (48%) had actively sought additional information about medical specialties, while 52% had not (Table 5).

**Table 5:** Level of awareness about various medical specialties and career pathways.

Variables		n (%)
Familiarity with medical specialty	Very familiar	27 (21)
	Somewhat familiar	65 (52)
	Not very familiar	34 (27)
	Not familiar at all	0
Actively sought information about specialties	Yes	61 (48)
	No	66 (52)
Awareness of career pathways and training requirements	Yes, very aware.	23 (18)
	Somewhat aware	44 (35)
	Not very aware	48 (38)
	Not aware at all	12 (9)

**Figure 8:**
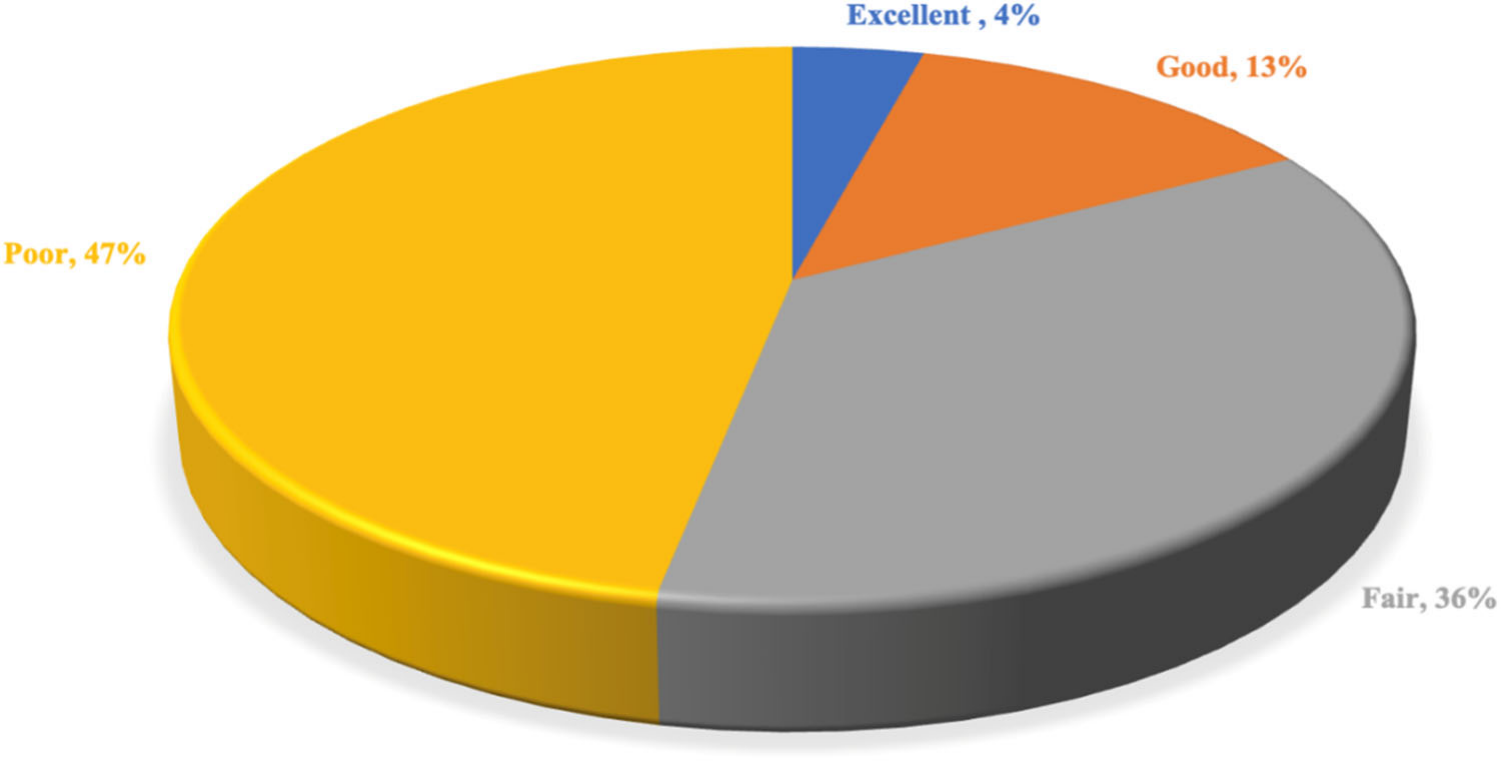
Availability of mentorship and informational sessions.

**Figure 9:**
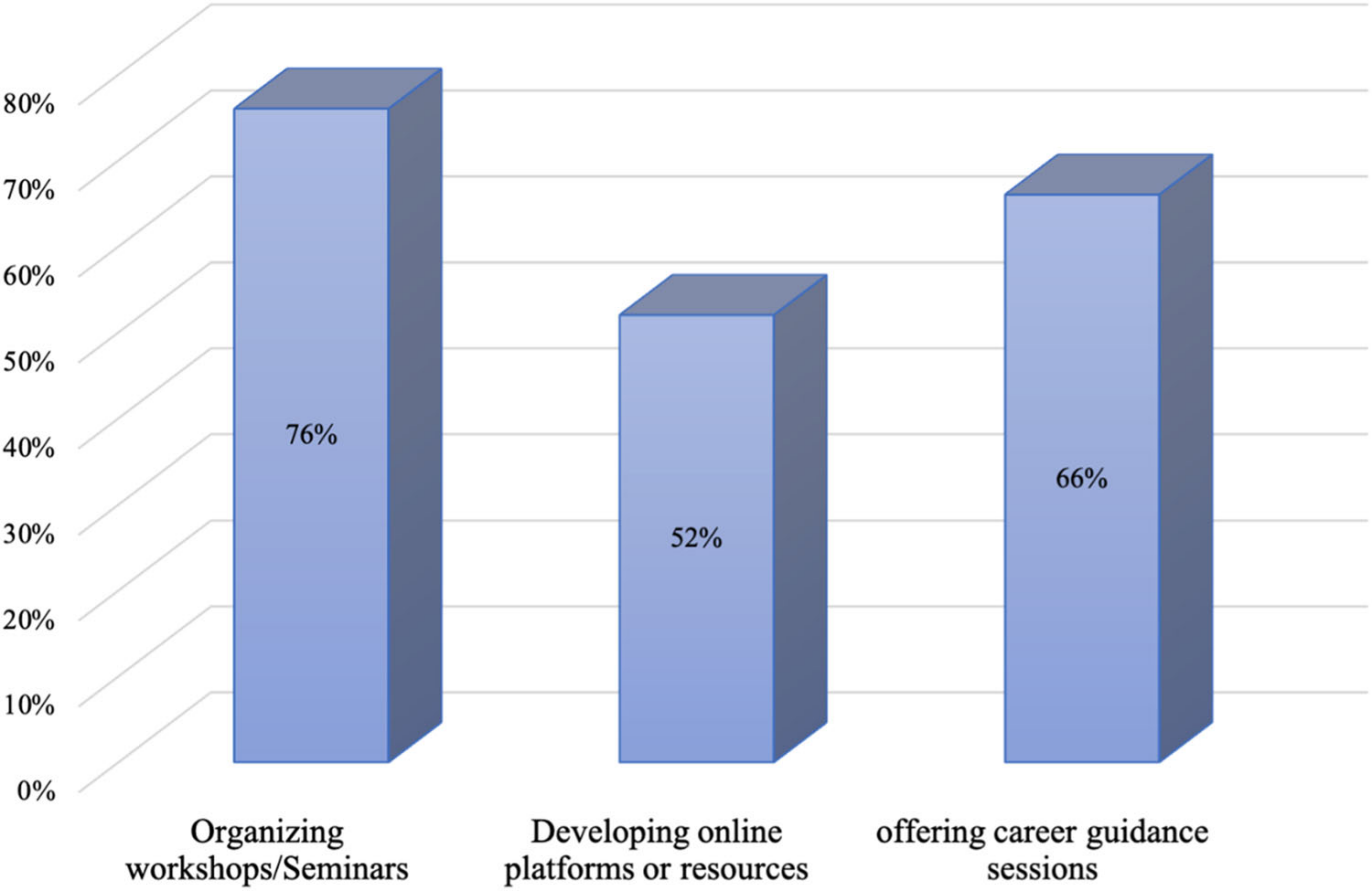
Measures to enhance awareness among medical students.

A majority of 83 percent of participants rated the availability of mentorship and informational sessions regarding specialization within their institutions as poor (47%) and fair (36%). Seventeen percent rated it as good (13%) and excellent (4%) (Figure 8). To enhance awareness, 76% suggested organizing workshops and seminars (Figure 9).

### 3.5 Future Plans

**Figure 10:**
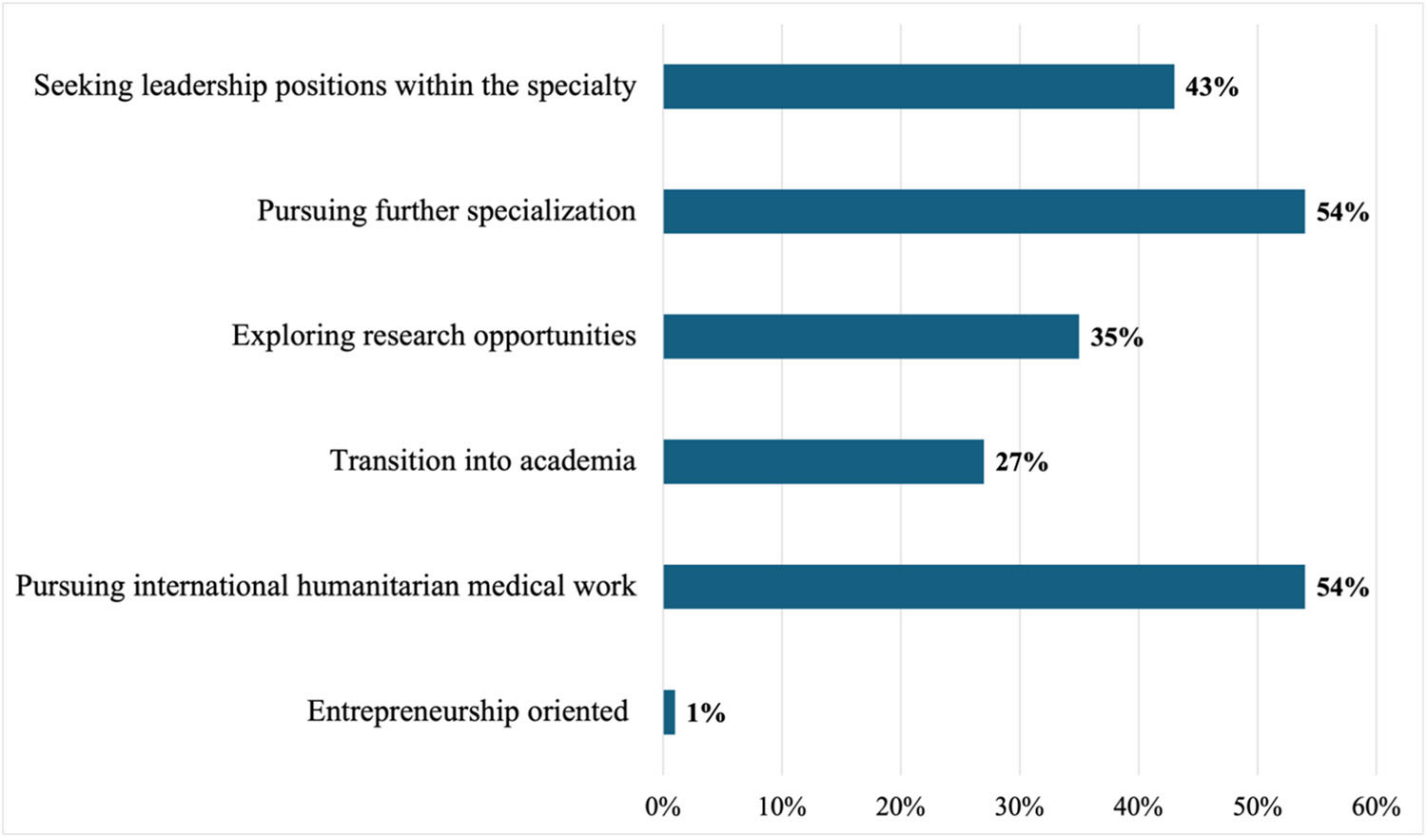
Future plans.

Fifty‐four percent (54%) plan to pursue further specialization and sub‐specialization in their chosen fields, international humanitarian medical work (54%), medical academics (27%), research (35%), and 1% expressed interest in future entrepreneurship (Figure 10).

## 4 Discussion

### 4.1 Demographics

In our study, 127 (66 (52%) males and 61(48%) females) medical students participated, with a 1.1:1 male‐to‐female ratio. This gender distribution is similar to findings from Botswana, where 60% of medical students were male and 40% female [14]. Conversely, a study in Sudan found a higher proportion of female participants (61.4%) compared to males (38.6%) [15]. The average age of participants in our study was between 20 and 25 years, consistent with the average age of 23.5 years reported by Rukewe et al. [14]. Notably, 3% of participants were over 35 years old, suggesting that most undergraduate medical students in Zambia are under 35, predominantly in their early 20s.

### 4.2 Specialty Preference

Internal medicine was the most preferred specialty among participants (12%), followed by cardiovascular surgery (10%) and obstetrics and gynecology (9%). We observe a difference in specialty preferences among males and females in the study. Among the male participants internal medicine, cardiothoracic surgery, obstetrics and gynecology and neurosurgery were the most common preferences whereas psychiatry, dermatology and pediatrics and child health were common among the females. Similar differences in preferences between male and females has been reported in the literature [16]. In a study by Khamees et al., surgical specialties were most preferred, and internal medicine was also popular, with 22% of participants undecided [17]. In Jordan, male students tended to choose surgical specialties, while females preferred medical specialties [18]. In Botswana, males favored surgical specialties and emergency medicine, with less interest in family medicine, anesthesia, public health, and basic medical sciences [14]. Conversely, female students in Saudi Arabia preferred surgery, pediatrics, and ophthalmology [19]. The choice of specialty often intersects with lifestyle considerations. Some studies have, however, found no gender influence on preference [20].

In this study, nonsurgical specialties were more popular 59.1% than surgical specialties 48.9%. Surgical specialties were more popular among male participants while nonsurgical specialties were more common among female participants similar to the study by Al‐Beitawi, Al‐Shatanawi et al. [18]. However, the difference was not statistically significant.

There was no statistically significant difference between surgical and nonsurgical specialties across all the age groups and years of study. Of note is that nonsurgical specialties were progressively more popular than surgical specialties with the biggest difference in the final year of study.

### 4.3 Factors Influencing

Personal interest was the dominant factor influencing specialty choice (74%), followed by work‐life balance. This aligns with findings from Alawad et al. [15], who also identified personal interest as a primary factor, consistent with studies from Saudi Arabia [19], Pakistan [7], and Taiwan [21, 22]. Levaillant et al. similarly reported that lifestyle and work‐life balance were significant considerations [23].

Prestige was a less dominant factor in our study (8%). Regarding financial considerations, 41% of participants indicated that potential earnings significantly influenced their choice, while 36% reported a moderate influence, 11% a minor influence, and 12% no influence. This contrasts with Khamees et al., who found that prestige, patient interaction opportunities, and high income were significant factors [17].

Mentor influence was reported by only 19% of participants. The importance of role models, particularly those of the same gender, is crucial in shaping career decisions. Female students, for example, may avoid surgical specialties due to a lack of female role models, opting instead for fields like pediatrics [24, 25]. Regarding job satisfaction and earning potential, 47% of participants believed their chosen specialty would provide a favorable balance, 37% were neutral, 12% thought it would be unfavorable, and 7% were unsure. Concerns about job availability and market saturation were noted by 54% of participants.

### 4.4 Level of Awareness Among Medical Students About Various Medical Specialties and Career Pathways

Awareness levels varied, with only 21% of participants very familiar with available medical specialties, 52% somewhat familiar, and 27% not very familiar. This suggests limited knowledge about the options available in medicine, potentially constraining their decisions. Despite 48% actively seeking information about various specialties, our results differ from other studies. In Sudan, 89.2% of participants had prior knowledge about their chosen specialties [15] and in Saudi Arabia, 68.6% reported adequate knowledge [19]. In the United States, career counseling services are recommended to help medical students select specialties aligned with their interests and skills [26].

The absence of good mentorship and information sessions was a major in this study with 83% of participants rating it as either poor (47%) or fair (36%). Only 17% consider mentorship availability good or excellent, indicating a need for improved support services. This is similar to a study by Edmund N. O. et al., who reported that 74.6% of students in southeast Nigeria had no form of career guidance during their stay in medical school [27]. Suggestions for enhancing awareness included organizing workshops/seminars (76%), developing online resources (52%), and offering career guidance sessions (66%). The need for mentorship in medical schools has been echoed in several other similar studies in different parts of the world including Nigeria [27, 28], Kenya [24], Gambia [29], Israel [30], and Saudi Arabia [19]. Mentorship will expose medical students to experiences of specialists in the desired fields allowing them to make more informed career choices [31, 32, 33, 34]. The use of social media has been proven as a cost‐effective tool in training, mentorship and practice in LMICs with the advantage of breaking the geographical barrier to mentorship [35]. This in the long run may translate into more fulfillment and improved healthcare delivery.

The limitations of the study include the small sample size of 127 participants which may reduce the generalizability of the study. This study relies on self‐reported data, which may introduce certain biases, such as social desirability bias and recall bias. Social desirability bias may have led respondents to answer questions in a manner they perceive as favorable, potentially affecting the accuracy of the results. Additionally, recall bias could influence participants' ability to accurately remember past events or behaviors, which may impact the reported findings. The cross‐sectional design of the study only captures the data at a single point in time. This limits the ability to draw conclusions about changes in the specialty preferences over the course of the clinical clerkship. Acknowledging these limitations helps contextualize the results and underscores the need for cautious interpretation.

## 5 Conclusion

This study identifies key challenges in specialty selection among medical students. Internal medicine, cardiovascular surgery, and obstetrics and gynecology emerged as the top specialty choices, influenced primarily by personal interest, work‐life balance, and financial considerations. Despite the acknowledged importance of mentorship, many students rated the availability of mentorship and informational resources as inadequate, highlighting a critical area for improvement. These findings underscore the necessity for enhanced mentorship programs, comprehensive career guidance, and targeted informational workshops to support students in making informed specialty choices, ultimately contributing to a more prepared and satisfied medical workforce.

## Author Contributions

**Gerald Musa:** conceptualization, writing – original draft, writing – review & editing, formal analysis, supervision. **Samuel Chilawa and Bipin Chaurasia:** writing – conceptualization, writing – original draft, writing – review & editing, formal analysis. **Alick Bwanga:** conceptualizing, reviewing, editing, supervision. **Bupe Mumba Mwela:** writing – original draft, writing – review & editing, formal analysis. **Laston Chikoya and Bipin Chaurasia:** writing – review & editing, proof reading and editing.

## Ethics Statement

Institutional Review Board (IRB) approval was not required due to the nature of the study. The research was observational, questionnaire‐based, involved no interventions, and did not collect sensitive personal data. Participation was entirely voluntary, and informed consent was obtained from all participants before data collection. The study adhered to ethical guidelines and the principles outlined in the Declaration of Helsinki.

## Consent

Informed, written consent was obtained from all participants included in the study.

## Conflicts of Interest

The authors declare no conflicts of interest.

### 1 Transparency Statement

The lead author Bipin Chaurasia affirms that this manuscript is an honest, accurate, and transparent account of the study being reported; that no important aspects of the study have been omitted; and that any discrepancies from the study as planned (and, if relevant, registered) have been explained.

## Data Availability

Data sharing is not applicable to this article as no new data were created or analyzed in this study. The data that support the findings of this study are available from the corresponding author upon reasonable request.
